# Efficient Synthesis of Steroidal Intermediates with a C17 Side Chain from Phytosterols by Genetically Modified *Mycolicibacterium neoaurum* NRRL B‐3805 Strain

**DOI:** 10.1002/open.202500086

**Published:** 2025-05-16

**Authors:** Xuemei Li, Liangyan Zhu, Qiong Wu, Rui Zhang, Yiyin Liu, Na Liu, Jinhui Feng, Qiaqing Wu, Dunming Zhu

**Affiliations:** ^1^ National Engineering Research Center of Industrial Enzymes and Tianjin Engineering Research Center of Biocatalytic Technology National Center of Technology Innovation for Synthetic Biology, and Tianjin Institute of Industrial Biotechnology Chinese Academy of Sciences Tianjin 300308 China; ^2^ University of Chinese Academy of Sciences Beijing 100049 China

**Keywords:** aldolases, biotransformations, carboxylic acid reductases, *Mycolicibacterium neoaurum*, phytosterols, steroidal intermediates

## Abstract

22‐Hydroxy‐23,24‐bisnorchol‐4‐ene‐3‐one (4‐HBC) and 3‐oxo‐4,17‐pregnadiene‐20‐carboxylic acid methyl ester (PDCE) are useful precursors for the synthesis of steroidal active pharmaceutical ingredients. In this study, we identify the sterol metabolism‐related genes, which encode the aldolases (Ltp2 and Thl) and carboxylic acid reductases (CAR) in *Mycolicibacterium neoaurum* NRRL B‐3805 (B3805), by analysis of the metabolites from phytosterols biotransformation. Based on these results, a genetically modified strain is constructed by disrupting the *kstD, ltp2,* and *hsd4A* genes and overexpressing the aldolase gene (*thl*) in the strain B3805. This recombinant strain (B3805V) is able to transform 5 g L^−1^ phytosterols to 2.0 g L^−1^ 4‐HBC without detectable AD by‐product. Additionally, by disrupting the *ltp2* and *car* genes, a strain (strain B3805VI) is obtained to transform phytosterols to PDCE with 1.44 g L^−1^ titer. The PDCE concentration is further increased by about 42% to 2.1 g L^−1^ without 4‐HBC by‐product by deleting *thl* gene (strain B3805VII). On the preparative scale, the strain B3805VII transforms 10 g L^−1^ of phytosterols into PDCE with 5.1 g L^−1^. This study presents one‐step bioproduction of pharmaceutically important 4‐HBC and PDCE with high yield and purity from bio‐renewable phytosterols, which are readily available as a by‐product from the plant oil industry.

## Graphical abstract

Two genetically modified strains are designed and constructed to produce 22‐hydroxy‐23,24‐bisnorchol‐4‐ene‐3‐one (4‐HBC) and 3‐oxo‐4,17‐pregnadiene‐20‐carboxylic acid methyl ester (PDCE) by disrupting and overexpressing the aldolase gene (*thl*) in the genetically modified strains of *Mycolicibacterium neoaurum* NRRL B‐3805, respectively, which are useful precursors for the synthesis of steroidal active pharmaceutical ingredients.

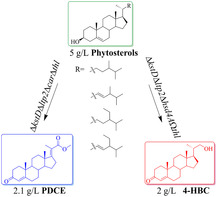

## 1 Introduction

The utilization of phytosterols as primary materials in the synthesis of steroidal medicines has gained popularity owing to their bio‐renewability and being readily available as a by‐product from vegetable oil industry.^[^ ^1^ ^]^ Microbial processes have been developed to efficiently break down the side chain of phytosterols, resulting in the industrial production of 19‐carbon steroidal intermediates such as 4‐androstene‐3,17‐dione (AD), androsta‐1,4‐en‐1,17‐dione (ADD), and 9α‐hydroxy‐androst‐4‐en‐1,17‐dione (9α‐OH‐AD).^[^ ^2^ ^]^ These intermediates serve as important building blocks for the commercial synthesis of various steroidal active pharmaceutical ingredients (APIs).

**Figure 1:**
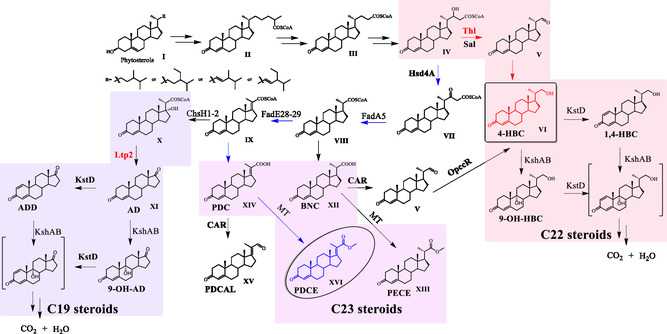
Overview of the cholesterol degradation pathway.,[^2^, ^10^, ^12^] Thl and Sal, aldolase; CAR, carboxylic acid reductases; MT, methyltransferase. 4‐HBC, 22‐hydroxy‐23,24‐bisnorchol‐4‐ene‐3‐one; PDCE, 3‐oxo‐4,17‐pregnadiene‐20‐carboxylic acid methyl ester; PECE, 3‐oxo‐4‐pregnaene‐20‐carboxylic acid methyl ester; PDCAL, 3‐oxo‐4,17‐pregadiene‐20‐carbaldehyde; BNC, 3‐oxo‐23,24‐bisnorchol‐4‐en‐22‐oate.

Over the past decades, a putative metabolic pathway has been proposed based on the intermediates identified in the sterol degradation. Phytosterols undergo degradation via two biochemical processes: elimination of alkyl side‐chain and catabolism of the core ring. The metabolism of the core rings is initiated by the transformation of 3β‐ol‐5‐en‐ to 3‐keto‐4‐en‐ moiety,^[^ ^3^ ^]^ and further mineralization of the steroidal core rings is proposed to employ a 9,10‐seco pathway, which was named due to the cleavage between C‐9 and C‐10 of the sterol ring.^[^ ^4^ ^]^ The initial terminal oxidation of alkyl side‐chain is believed to employ the steroid C27 monooxygenases (cyp125 and cyp142) of cytochrome P450s.^[^ ^5^ ^]^ A β‐oxidation‐like mechanism is suggested for the further degradation of the alkyl side‐chain. This process may involve several enzymes, including sterol‐CoA ligases such as FadD19 and FadD17,^[^ ^6^ ^]^ dehydrogenases including ChsE1‐5, FadE26‐29,34, or Scd1‐2,^[^ ^7^ ^]^ hydratases like ChsH1‐2 or Shy,^[^ ^8^ ^]^ acyl‐CoA thiolase such as FadA5,^[^ ^9^ ^]^ β‐hydroxyacyl‐CoA dehydrogenase (Hsd4A),^[^ ^10^ ^]^ and aldolase such as Ltp2‐4 or Sal1‐2.[^8^, ^11^] Manipulation of this degradation pathway has resulted in valuable strains for the production of C‐19 and C‐22 steroidal building blocks (**Figure** **1**).[^2^, ^10^, ^12^]

**Figure 2:**
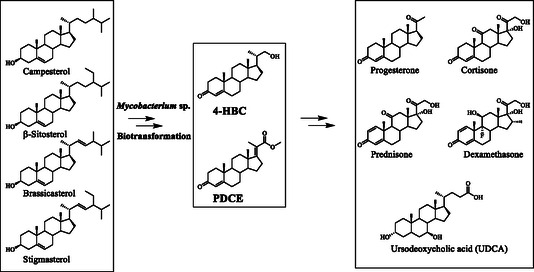
The key C22 intermediates for the synthesis of various types of steroidal active pharmaceutical ingredients with a side‐chain at C17.[^12^, ^13^]

Many steroidal APIs, including progestational and adrenocortical hormones, possess a side‐chain at C17, which was typically constructed by introducing a nucleophile at C17 carbonyl group, followed by subsequent transformations.[^12^, ^13^] Recently, the transformations of phytosterols into 22‐hydroxy‐23,24‐bisnorchol‐4‐ene‐3‐one (4‐HBC, VI), 22‐hydroxy‐23,24‐bisnorchol‐1,4‐dien‐3‐one (1,4‐HBC), and 9,22‐dihydroxy‐23,24‐bisnorchol‐4‐ene‐3‐one (9‐OH‐HBC), wherein a 3‐carbon isopropanol side chain exists at C17‐position, have been studied.^[^ ^10^, ^12^, ^14^ ^]^ The Hsd4A and a reductase (mnOpccr) might be involved in the formation of C22 steroids.^[^ ^10^, ^12^ ^]^ The deletion of *hsd4A* gene in NwIB‐XII resulted in a 40% molar yield of 1,4‐HBC, while deletion of *hsd4A* gene in NwIB‐XIIΔ*kstD123* led to a 49% molar yield of 4‐HBC.^[^ ^10^ ^]^ Inactivation of the *hsd4A* and overexpression of mnOpccr in *Mycolicibacterium neoaurum* CCTCC AB2019054 achieved a 93% conversion of phytosterols to 4‐HBC.^[12b]^ These C22 steroids offer new building blocks for the synthesis of steroidal APIs with a C17‐side chain. Recently, formation of 9‐OH‐PDC (9α‐hydroxy‐3‐oxo‐4,17‐pregnadiene‐20‐carboxylic acid) and 9‐OH‐PDCE (the methyl‐esterified 9‐OH‐PDC) as C22 steroid intermediates from phytosterols has also been reported.[^11^, ^15^] Arima et al. reported that the *Nocardia corallina* IFO 3338 degraded cholesterol, resulting in the accumulation of pregn‐1,4‐dien‐3‐one‐20‐carboxylic acid (1,4‐BNC, as Δ^1^ BNC).^[^ ^16^ ^]^ Owen et al. confirmed *Pseudomonas* sp. NCIB 10590 possessed the ability to degrade cholesterol to form both 1.4‐BNC and BNC.^[^ ^17^ ^]^ A mutant strain of *Rhodococcus rhodochrous* RG32 has been reported to degrade cholesterol predominantly accumulating 1,4‐BNC.^[^ ^6^ ^]^ The genome‐modified strains of *Mycobacterium neoaurum* have been constructed to degrade phytosterols, generating 3‐oxo‐4,17‐pregnadiene‐20‐carboxylic acid methyl ester (PDCE) and PECE, or 9‐OH‐PDCE, respectively,[^11^, ^12^, ^15^] and 9α‐hydroxy‐3‐oxo‐4,17‐pregnadiene‐20‐carboxylic acid methyl ester (9‐OH‐PDCE) has been prepared at gram scale.^[11d]^ 4‐HBC and PDCE would be useful intermediates for the synthesis of steroidal APIs because the C17‐C20 double bond, a side chain at C17, and the ester groups offer multiple sites for the construction of the molecular skeleton and functionalization (**Figure** **2**).^[^ ^13^ ^]^ However, the production of 4‐HBC or PDCE encounters certain challenges. A variety of by‐products, such as 9‐OH‐PDC, were also formed during the degradation of phytosterols by the available microbial strains, thus leading to the difficulty in production isolation and low yield of the desired products. This is because the intricate and indistinct sterol biodegradation pathways present a barrier to constructing effective strains for the production of the target compounds with high purity.

In this study, we performed an investigation on the biosynthesis of 4‐HBC and PDCE from phytosterols. Manipulation of β‐hydroxyacyl‐CoA dehydrogenase (Hsd4A) and aldolase (Ltp2 or Thl) in the *M. neoaurum* NRRL B‐3805 (B3805) resulted in 4‐HBC as the main product instead of AD. The PDCE‐producing strain was then constructed by knocking out both aldolase and carboxylic acid reductase (CAR), and PDCE was prepared from phytosterols in an eco‐friendly and efficient manner.

## 2 Results and Discussion

### 2.1 Identification and Classification of *ltp2* in B3805

*M. neoaurum* NRRL B‐3805 (B3805) is capable of metabolizing phytosterols to produce AD as main product, as well as androst‐1,4‐dien‐3,17‐dione (ADD) and 4‐HBC as by‐products.^[^ ^18^ ^]^ In our previous studies, methyl 3‐oxo‐23,24‐bisnorchol‐4‐en‐22‐oate (PECE, **XIII**, Figure 1) was identified as a by‐product in the metabolism of phytosterols by strain B3805 and knockout of the 3‐ketosteroid‐Δ^1^ ‐dehydrogenase gene (*kstD*, protein ID AMO08643.1) prevented the production of ADD.^[5d]^ A protein complex of Ltp2 with a DUF35 domain derived from the C‐terminal domain of a hydratase (ChsH2_DUF35_) was reported to be able to cleavage the C22 intermediate to generate AD through a reverse aldol mechanism. The strain of B3805 was sequenced (GenBank accession number CP011022^[^ ^19^ ^]^), and a putative *ltp2* gene (the accession number of AMO08652.1) was identified. The *ltp2* gene was deleted in the *kstD*‐deletion B3805 strain (B3805Δ*kstD*, B3805I) by two‐step homologous recombination system.^[^ ^20^ ^]^ The genetically modified strain (B3805Δ*kstD*Δ*ltp2*, B3805II) was confirmed by polymerase chain reaction (PCR) using primers (*ltp2*_3805del_‐U‐Fand *ltp2*_3805del_‐D‐R) and sequencing (Figure S1, Supporting Information).

**Figure 3:**
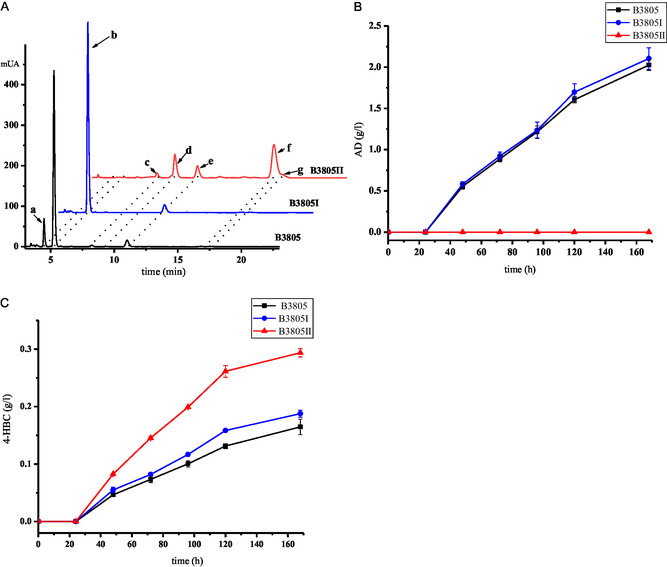
Metabolite analyses of phytosterols transformed by B3805, B3805I (B3805Δ*kstD*), and B3805II (B3805Δ*kstD*Δ*ltp2*) strains. (A) High‐performance liquid chromatography analysis of B3805, B3805I, and B3805II cultured with 5 g L^−1^ phytosterol. (a) ADD; (b) AD (XI); (c) unknown product; (d) XV; (e) 4‐HBC; (f) XVI; (g) XIII. (B) Time course of AD accumulation by B3805, B3805I, and B3805II strains. (C) Time course of 4‐HBC accumulation by B3805, B3805I, and B3805II.

The transformations of phytosterols were carried out by the genome‐modified strains. The cultures were extracted with ethyl acetate and then analyzed by high‐performance liquid chromatography (HPLC) as reported previously.^[^ ^21^ ^]^ The strain B3805I produced 2.1 g L^−1^ AD and 0.18 g L^−1^ 4‐HBC when 5.0 g L^−1^ phytosterols were served as substrate. However, no AD was detected, and only 0.3 g L^−1^ 4‐HBC was found in the cultures of the B3805II strain (**Figure** **3**B,C). This confirmed that putative *ltp2* gene encoded an aldolase for the cleavage of C22 intermediate to form AD through an aldol cleavage mechanism in B3805 strain. In comparison to strain B3805I, strain B3805II produced several additional products (**c**, **d**, and **f**, as shown in Figure 3A), along with 4‐HBC (**e**) and product **g**. The product **d** was analyzed by liquid chromatography–mass spectrometry (LC–MS) with *m/z* of 327.2297 [M + H]^+^ (Figure S2A, Supporting Information) and was speculated as 3‐oxo‐4,17‐pregadiene‐20‐carbaldehyde (PDCAL, **XV**) (Figure 1). The product **f** was purified and characterized as 3‐oxo‐4,17‐pregadiene‐20‐carboxylic acid methyl ester (PDCE, **XVI**, Figure 1) through LC–MS with an *m/z* of 357.2401 [M + H]^+^ and ^1^H, ^13^C NMR in chloroform‐d (Figure S2B, and S3, Supporting Information). Product **g** was determined as methyl 3‐oxo‐23,24‐bisnorchol‐4‐en‐22‐oate (PECE, **XIII**, Figure 1).^[^ ^21^ ^]^ The product **c** was not identified. These intermediates were possibly generated through successive thioester hydrolysis, carboxylic acid reduction, or carboxyl methylation of the CoA intermediates VIII or IX, respectively (Figure 1). The compound X should be accumulated after the knockout of *ltp2*. However, the compound X and AD were not detected after knocking out Ltp2 in strain B3805I. We speculate that the compound X was able to dehydrate to form compound IX when Ltp2 was inactivated. Then, compound IX was transformed into compound XIV by thiolase, and compound XIV was converted into PDCE by methyltransferase.

### 2.2 Enhancement of 4‐HBC Accumulation by Overexpressing *thl* and Deleting *hsd4A* gene

In *Pseudomonas* sp. strain Chol1, an aldehyde intermediate was discovered during the degradation of cholate, and a steroid aldolase enzyme called Sal was identified as the catalyst for the retro‐aldol cleavage reaction, which involves the aldolytic cleavage of the steroid compound.[^8^, ^22^] In the *M. neoaurum* strains, an aldolytic cleavage and a reductive reaction were proposed to produce HBCs.^[^ ^10^ ^]^ An aldolase *thl* was predicted to remove the acyl side chain from C24 intermediate to generate 4‐HBC.[^2^, ^12^] In the *M. smegmatis* strain, deletion of aldolase gene increased the production yield of AD by avoiding the formation of by‐product 4‐HBC.^[2b]^ Moreover, introduction of *thl* into the genetically modified strains of *Mycobacterium fortuitum* resulted in generation of HIP‐IPA in the biotransformation of phytosterols.^[^ ^23^ ^]^ Based on these findings, introduction of *thl* gene into B3805II strain and deletion of *hsd4A* gene would be capable of enhancing the formation of 4‐HBC.

**Figure 4:**
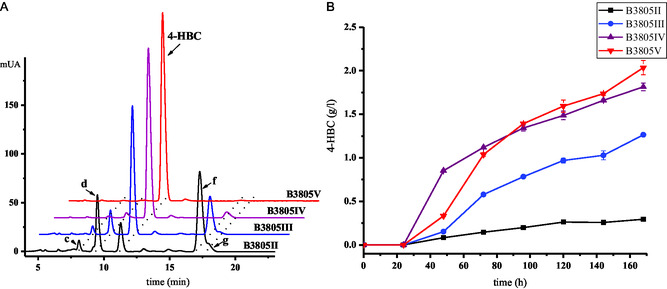
Metabolite analyses of phytosterols transformed by B3805II (B3805Δ*kstD*Δ*ltp2*), B3805III (B3805Δ*kstD*Δ*ltp2*Ω*thl*), B3805IV (B3805Δ*kstD*Δ*ltp2*Δ*hsd4A*), and B3805V (B3805Δ*kstD*Δ*ltp2*Δ*hsd4A*Ω*thl*) strains. (A) High‐performance liquid chromatography analysis of B3805II, B3805III, B3805IV, and B3805V strains cultured with 5 g L^−1^ phytosterol. **c**, unknown product; **d**, XV; **f**, XVI; **g**, XIII. (B) Time course of 4‐HBC accumulation by B3805II, B3805III, B3805IV, and B3805V strains.

**Scheme 1:**
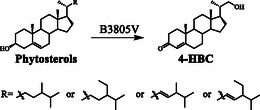
Biotransformation of phytosterols to 4‐HBC by B3805V strain

As such, both of *thlA* (AMO08280.1) and *thlB* (AMO05741.1) were overexpressed, and *hsd4A* gene (AMO07649.1) was knocked out in B3805II strain, resulting in the strains B3805III (B3805Δ*kstD*Δ*ltp2*Ω*thl*), B3805IV (B3805Δ*kstD*Δ*ltp2*Δ*hsd4A*), and B3805V (B3805Δ*kstD*Δ*ltp2*Δ*hsd4A*Ω*thl*). The transformations of phytosterols were carried out using these strains, and the reaction mixtures were extracted and analyzed by HPLC.^[^ ^21^ ^]^ As shown in **Figure** **4** and **Scheme** **1**, the strains B3805III and B3805IV produced 1.22 and 1.79 g L^−1^ of 4‐HBC, respectively, when 5.0 g L^−1^ phytosterols served as the substrate. The B3805V strain yielded 2.00 g L^−1^ 4‐HBC without detected by‐products, which was 64% and 12% higher than those of strains B3805III and B3805IV, respectively. Therefore, the yield of 4‐HBC was improved from 0.3 g L^−1^ (strains B3805II) to 2.00 g L^−1^ by overexpressing *thl* and inactivating *hsd4A*, and no AD was detected. The concentration of 4‐HBC was not increased by overexpressing of *thlA* or *thlB* alone (data not shown). Although it was reported that deletion of Ltp2 in genetically modified *M. neoaurum* ATCC 25795 was still able to generate AD,^[2c]^ in our hands, the abrogation of aldolase Ltp2 in B3805 strain blocked the sterol side chain degradation to generate AD, while the aldolases (*thl*) are the key functional enzymes for the formation of 4‐HBC. This demonstrated an interesting strategy for improving the concentration and purity of 4‐HBC by manipulating the strain with different aldolase enzymes of distinct substrate specificity.

### 2.3 Enhancement of PDCE Accumulation by Deleting *thl* and *Car*

The methyl ester as C23 steroid intermediates has been identified as a by‐product in wild‐type strain B3805.^[^ ^21^ ^]^ Recently, the C23 steroid intermediates were successfully accumulated in *M. neoaurum* with *chsH* and/or *ltp2* deficiency.[^11^, ^12^, ^15^] It has been reported that the strain was constructed to produce C22 steroid drug precursor 9‐OH‐PDCE by inactivating *kstDs* and *ltp2*, and overexpressing *hsd4A* and *chsE1‐chsE2* at the same time in *M. neoaurum* ATCC 25 795.^[11d]^ Similarly, a study with *M. neoaurum* DSM 44704 also achieved the formation of PDCE by knocking out *kshA*.[^12^, ^15^] Generally, disruption of *ltp2* or *chsH* in vivo should generate C23 steroid intermediates as the major product, such as PDCE or 9‐OH‐PDCE. In our previous study, two carboxylic acid reductase genes (*car1* and *car2*) were found to be involved in the degradation of HIP, and the strains Δ*fadD3*Δ*car1,2* and Δ*fadE30*Δ*car1,2* were able to efficiently transform phytosterols to HIP and HIL, respectively.^[^ ^24^ ^]^ Because 3‐oxo‐4,17‐pregadiene‐20‐carbaldehyde (PDCAL, **XV**) was detected in the transformation of phytosterols by strain B3805II (Figure 3A(d)), CAR was hypothesized to be involved in the metabolic pathways of compound **XII** and/or **XIV**.

**Figure 5:**
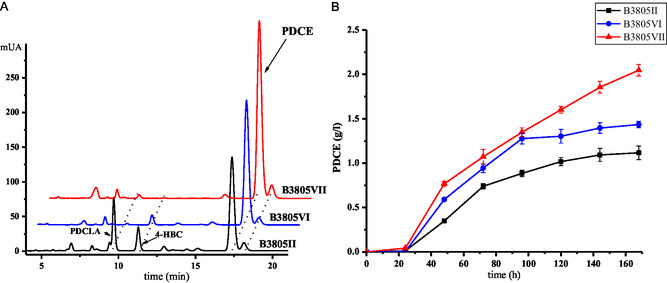
Metabolite analyses of phytosterols transformed by B3805II (B3805Δ*kstD*Δ*ltp2*), B3805VI (B3805Δ*kstD*Δ*ltp2*Δ*car*), and B3805VII (B3805Δ*kstD*Δ*ltp2*Δ*car*Δ*thl*). (A) High‐performance liquid chromatography analysis of metabolites by B3805II, B3805VI, and B3805VII strains cultured with 5 g L^−1^ phytosterol. (B) Time course of PDCE accumulation by B3805II, B3805VI, and B3805VII strains.

**Scheme 2:**
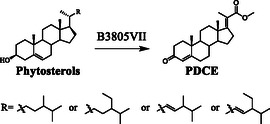
Biotransformation of phytosterols to PDCE by B3805VII strain

Therefore, the CAR (ID: AMO03891.1), which had an identity of 71% and 69% with CAR1 and CAR2 of *Mycobacterium fortuitum* (ATCC 6841), respectively, was inactivated in strain B3805II to give the strain B3805VI (B3805Δ*kstD*Δ*ltp2*Δ*car*). As shown in **Figure** **5**, when the *car* gene was knocked out, the production of PDCAL (**XV**) was extremely declined, while the concentration of PDCE (**XVI**) was increased to 1.44 g L^−1^. Additionally, a minimal amount of 4‐HBC was found from the transformation of phytosterols with strain B3805VI. To further enhance PDCE production and eliminate the formation of 4‐HBC, the *thl* gene clusters were knocked out to obtain strain B3805VII (B3805Δ*kstD*Δ*ltp2*Δ*car*Δ*thl*). As shown in Figure 5 and **Scheme** **2**, when the *thl* gene clusters were knocked out, the 4‐HBC production was blocked, and the accumulation of PDCE was enhanced to a concentration of 2.05 g L^−1^, 42.4% higher than strain B3805VI (1.44 g L^−1^).

The inactivation of *car* genes may eliminate the by‐products and improve the accumulation of the desired product PDCE or 3‐oxo‐4,17‐pregnadiene‐20‐carboxylic acid (PDCA, **XIV**, Figure 1). Considering that the carboxylic acid was the substrate for both carboxylic acid reductase and methyltransferases, inactivation of carboxylic acid reductase favored the formation of the methyl ester of PDCA, which can be generated by an O‐methyltransferase using SAM as the methyl group donor.^[^ ^25^ ^]^ Therefore, the strain with inactivation of *ltp2*, *car,* and *thl* (B3805VII) produced PDCE in high yield from phytosterols (Figure 5), a readily available renewable starting material.

Considering the unique structure of PDCE, which has different biological activities and applications from C19 steroids, it is worth further exploring its biological production. Therefore, the PDCE was prepared at a pilot scale by carrying out the transformation of 10 g L^−1^ of phytosterols by B3805VII for 7 days in a 3 L fermentation. The fermentation bioconversion mixture was extracted with ethyl acetate and analyzed by HPLC. ≈5.1 g L^−1^ of PDCE was achieved without PDCAL and 4‐HBC due to deletion of CAR and *thl*, although along with a small amount of PECE in the bioconversion mixture.

## 3 Conclusion

In summary, we developed genetically modified strains capable of efficiently producing 4‐HBC and PDCE. By eliminating the genes of *ltp2* and *hsd4A* and enhancing the expression of the *thl* gene, 2.0 g L^−1^ 4‐HBC was generated from 5 g L^−1^ of phytosterols without the unwanted by‐product AD. Furthermore, the generation of PDCE was systematically studied. The strain B3805VI with the deletions of *ltp2* and *car* showed a 30% increase in PDCE production with minimal 4‐HBC by‐product. Knocking out *thl* in strain B3805VI further increased the PDCE production to about 2.1 g L^−1^ without any detectable 4‐HBC by‐product in the extraction of the culture. These findings have led to the development of highly efficient processes for the production of 4‐HBC and PDCE with exceptional yield and purity. Furthermore, these results provide valuable insights for the molecular engineering of industrial strains for the production of new intermediates, which may be used in the design of environmentally friendly and commercially viable routes for steroidal drug synthesis.

## 4 Experimental Section

4.1

4.1.1

##### Chemicals and Strains

The phytosterols (95%), AD, 4‐HBC, and other chemical reagents and solvents were purchased from chemical companies with reagent grade or the highest purity available. Phusion high‐fidelity DNA polymerase and FastDigest restriction enzymes were purchased from Fermentas (ThermoFisher, USA). The plasmid extraction kit and gel extraction kit were supplied by SIGMA (Beijing, China). The T5 Direct PCR kit (plant) and the CloneExpress II/MultiS One Step Cloning Kit were from Tsingke (Beijing, China) and Vazyme (Nanjing, China), respectively.

The strains used in this study are listed in **Table** **1**. Strain B3805Δ*kstD* (B3805I) was a genetically modified strain of B3805 with the unmarked deletions of one 3‐ketosteroid‐Δ^1^‐dehydrogenase gene *(kstD*).

**Table 1:** Strains and plasmids used in this study.

Straina)	Genotype and/or description	Source or reference
*Mycolicibacterium neoaurum* B‐3805		[19]
B3805I	*kstD*‐deleted strain of B3805	[5d]
	AD as the main product, none of ADD	
B3805II	*ltp2* deleted in B3805I strain none of AD	This study
B3805III	B3805II strain harboring plasmid pMV261‐*thl*	This study
B3805IV	*hsd4A* deleted in B3805II strain	This study
B3805V	B3805IV strain harboring plasmid pMV261‐*thl*	This study
B3805VI	*car* deleted in B3805II strain	This study
B3805VII	*thl* deleted in B3805VI strain	This study
Plasmids		
p2NIL	Vector of two homologous arms for allelic recombination in mycobacteria, *Kan* ^R^	[20]
pGOAL19	*Hyg* ^*R*^, Pag85‐*lacZ*, P_*hsp60*_‐*sac*B, *Pac*I cassette vector, *Amp* ^*R*^	[20]
pKH_del_‐*ltp2*_3805_	p2NIL Harboring two homologous arms of *ltp2* with selection cassette of pGOAL19 for deletion in mycobacteria	This study
pKH_del_‐*car*_3805_	p2NIL Harboring two homologous arms of *car* with selection cassette of pGOAL19 for deletion in mycobacteria	This study
pKH_del_‐*thl*_3805_	p2NIL Harboring two homologous arms of *thl* with selection cassette of pGOAL19 for deletion in mycobacteria	This study
pKH_del_‐*hsd4A*_3805_	p2NIL Harboring two homologous arms of *hsd4A* with selection cassette of pGOAL19 for deletion in mycobacteria	This study
pMV261	*Mycobacterium/Escherichia. coli* shuttle vector harboring hsp60 promoter, *Kan* ^*R*^	[26]
pMV261‐*thl*	pMV261 contain *thl* from *M. neoaurum* B‐3805	This study

a) *Kan* ^*R*^, kanamycin‐resistant; *Amp* ^*R*^, ampicillin‐resistant; *Hyg* ^*R*^, hygromycin‐resistant.

##### Gene Deletion and Expression

The unmarked gene deletion strains of B3805 were constructed using homologous recombination with the plasmids pGOAL19 and p2NIL, as described previously.^[^ ^20^, ^23^ ^]^ Two 1 kbp fragments were amplified from B3805 genomic DNA with pairs of primers (Table S1, Supporting Information) *ltp2*_3805_‐U‐F, *ltp2*_3805_‐U‐R, *ltp2*_3805_‐D‐F, *ltp2*_3805_‐D‐R, *car*_3805_‐U‐F, *car*_3805_‐U‐R, *car*_3805_‐D‐F, *car*_3805_‐D‐R, *thl*_3805_‐U‐F, *thl*_3805_‐U‐R, *thl*_3805_‐D‐F, *thl*_3805_‐D‐R, *hsd4A*_3805_‐U‐F, *hsd4A*_3805_‐U‐R, *hsd4A*_3805_‐D‐F, and *hsd4A*_3805_‐D‐R, respectively. These fragments were then ligated with linear p2NIL (digest with *Kpn*I and *Pst*I) and pGOAL19 (digest with *Pac*I) to produce the recombinant plasmid pKH_del_‐*ltp2*_3805_, pKH_del_‐*car*_3805_, pKH_del_‐*thl*_3805,_ and pKH_del_‐*hsd4A*_3805_ using the CloneExpress MultiS One Step Cloning Kit. The plasmids were introduced into the strains through electroporation, respectively. Then, the gene deletion strains were selected using a two‐step selection process and confirmed through PCR and gene sequencing.

The thiolase gene was cloned from B3805 by PCR using the primers *thl*_3805_‐F, *thl*_3805_‐R (Table S1, Supporting Information). These fragments and the plasmid pMV261^[^ ^26^ ^]^ were digested with *EcoR*I and *Hind*III and then ligated using a ClonExperss II One Step Cloning Kit to produce expression plasmid pMV261‐*thl* (Table 1). The plasmid was then transferred into strains by electroporation, and the strains hosting the expression plasmid were selected with 50 μg mL^−1^ kanamycin.

##### Biotransformation of Phytosterols and Product Analysis

For the biotransformation of phytosterols, all Mycolicibacteria strains were cultured as previously described.^[^ ^21^, ^24^ ^]^ Briefly, the strains were cultured in Luria‐Bertani (LB) broth with 0.5% Tween 80 at 30 °C for 2 days. Then, they were inoculated into a transformation culture containing defatted soy flour (10 g L^−1^), corn steep power (5 g L^−1^), (NH_4_)_2_HPO_4_ (2 g L^−1^), and emulsified phytosterols (5 g L^−1^), adjusted to pH 7.5 using NaOH.^[^ ^24^ ^]^ These strains were initially cultured at 30 °C for 20 h, followed by at 42 °C for 30 min for inducing the expression of thiolases. Subsequently, the strains were cultured at 30 °C for an additional 6 days. Culture samples (1 mL) were taken every 24 h (24, 48, 72, 96, 120, 144, and 168 h) to monitor the concentration of products (AD, 4‐HBC and PDCE) using HPLC.

For the preparative scale biotransformation, the B3805VII strains were cultured in seed medium with glucose (6 g L^−1^), yeast powder (15 g L^−1^), NaNO_3_ (5.4 g L^−1^), glycerol (2 g L^−1^), and NH_4_H_2_PO_4_ (0.6 g L^−1^), adjusted to pH 7.5, at 30 °C for 3 days. Then, the strain was inoculated into a transformation culture containing emulsified phytosterols (10 g L^−1^) at 30 °C for 7 days.

For the product analysis, the bioconversion mixture was extracted with 3 volumes of ethyl acetate, and analyzed by HPLC as previously described.^[^ ^21^ ^]^ The HPLC was equipped with a C18‐column (250 mm × 4.6 mm × 0.5 μm, Agilent, USA), and a mixture of methanol and water (80:20, v/v) was used as mobile phase at a flow rate of 0.8 mL min^−1^. Signals were detected with a diode array detector at wavelengths of 210, 230, and 254 nm.

##### Isolation and Identification of the Products by B3805II Strain

The culture of *ltp2* deletion strain (B3805II strain) was extracted with ethyl acetate, and the organic phase was concentrated under reduced pressure. The product was isolated and purified using a silica gel column with petroleum ether and ethyl acetate (6:1, v/v) as eluents. Preparative reverse phase recycling HPLC (Agilent 1260) with C18‐column was used for the final purification, using a mixture of methanol and water (70:30, v/v) at flow rate of 11 mL min^−1^. The detecting wavelengths were set at 254 and 210 nm. The ^1^H and ^13^C NMR spectra of PDCE were recorded at 400 MHz using Bruker Avance III devices with solvent CDCl_3_.

## Conflict of Interest

The authors declare no conflict of interest.

## Supporting information

Supplementary Material

## Data Availability

The data that support the findings of this study are available in the supplementary material of this article.
