# Supplementary material for: Efficient Synthesis of Steroidal Intermediates with a C17 Side Chain from Phytosterols by Genetically Modified Mycolicibacterium neoaurum NRRL B‐3805 Strain
Source: ChemistryOpen. 2025 May 16;14(9):e202500086. doi: 10.1002/open.202500086 (PMC12409849; doi:10.1002/open.202500086)
Supplement: Supplementary file 1 — Supplementary Material [file OPEN-14-e202500086-s001.pdf]

TABLE S1 Primers list used in this study

Primer name	sequence
hsd4A$_{3805del}$-U-F	ACGTTGTTGCCATTGCTGCAGCGGGTTGACACC CTGAAGGAAC
hsd4A$_{3805del}$-U-R	GAAAGGTTGCCGTCGTGACCGGCCACGCTCGGT GACTTCTTTG
hsd4A$_{3805del}$-D-F	CAAAGAAGTCACCGAGCGTGGCCGGTCACGAC GGCAACCTTTC
hsd4A$_{3805del}$-D-R	GTACCGCGGCCGCTTAATTAACTCGGAATAGCC GATGGAGAAG
Ltp2$_{3805del}$-U-F	ACGTTGTTGCCATTGCTGCAGGACCGACGGTCT GCATGAGCTG
Ltp2$_{3805del}$-U-R	CGATGGATTCCAACCTGGAGAGTACTATCGAGA TCGGCGGGAAGCTAC
Ltp2$_{3805del}$-D-F	AGCTTCCCGCCGATCTCGATAGTACTCTCCAGG TTGGAATCCATCGTG
Ltp2$_{3805del}$-D-R	GTACCGCGGCCGCTTAATTAACAAGTTCTTCTG GGATGGCGTC
car$_{3805del}$-U-F	ACGTTGTTGCCATTGCTGCAGAGCAGTGAGGCC TGCCGTTG
car$_{3805del}$-U-R	GACACAGCTCAACTTCGTCCCAGTACTGGGAAC TGGCAGACAAACACCTG
car$_{3805del}$-D-F	GGTGTTTGTCTGCCAGTTCCCAGTACTGGGACG AAGTTGAGCTGTGTCGG
car$_{3805del}$-D-R	GTACCGCGGCCGCTTAATTAACGCCGAAAATCT TGATGACCAGC
thl$_{3805del}$-U-F	ACGTTGTTGCCATTGCTGCAGGTACTACGGGGC GTCCAGCTTCC
thl$_{3805del}$-U-R	CGAAGTCCGTGGCCAGGGTGAGTACTTGTGCGA CGGCGAAGCTGACCAG
thl$_{3805del}$-D-F	GTCAGCTTCGCCGTCGCACAAGTACTCACCCTG GCCACGGACTTCG
thl$_{3805del}$-D-R	GTACCGCGGCCGCTTAATTAAGCGACGACTACA CGACCTTTTCC
thl$_{3805}$-F	GCGGATCCAGCTGCAGAATTCATGGGTTTGCGT GGTGACG
thl$_{3805}$-R	TACGTCGACATCGATAAGCTTCTATTCGGCGGC GGTGTAGTG

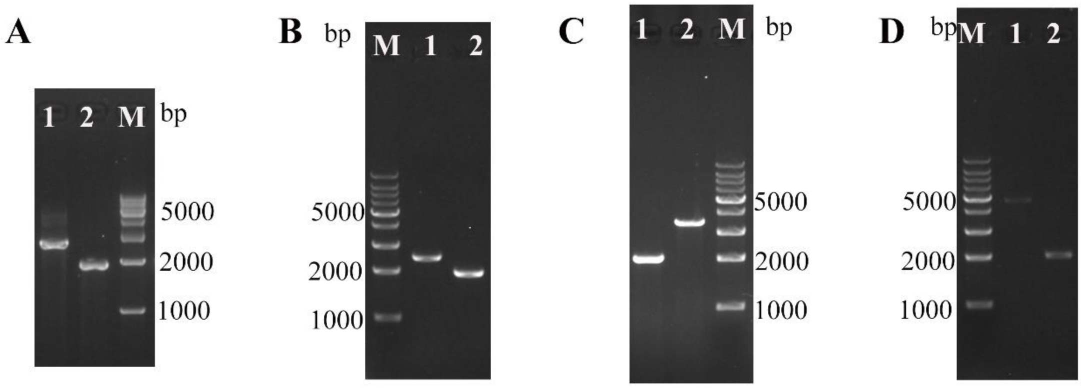

FIGUER S1 Identification of *ltp2, hsd4A, car and thl* genes deleted mutants of B3805I by PCR.

(**A**) Identification of *ltp2* gene deleted mutants of B3805I by PCR. Lanes M: DNA markers; 1: the PCR products of *ltp2* using B3805 as the control; 2: the PCR products of *ltp2* with a shortened size. (**B**) Identification of *hsd4A* gene deleted mutants of B3805II by PCR. Lanes M: DNA markers; 1: the PCR products of *hsd4A* using B3805 as the control; 2: the PCR products of *hsd4A* with a shortened size. (**C**) Identification of *car* gene deleted mutants of B3805II by PCR. Lanes M: DNA markers; 1: the PCR products of *car* with a shortened size; 2: the PCR products of *car* using B3805 as the control. (**D**) Identification of *thl* gene deleted mutants of B3805VI by PCR. Lanes M: DNA markers; 1: the PCR products of *thl* using B3805 as the control; 2: the PCR products of *thl* with a shortened size.

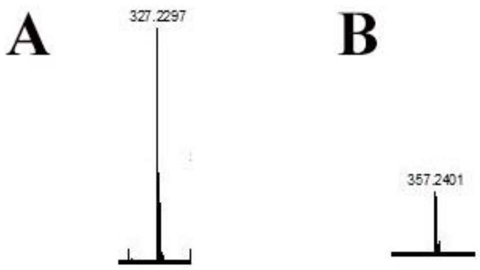

FIGURE S2 LC-MS of compound **d** and **f**.

(**A**) LC-MS of compound **d**, which was speculated as 3-oxo-4,17-pregadiene-20-carbaldehyde (PDCAL, **XV**). (**B**) LC-MS of compound **f** (**XVI**).

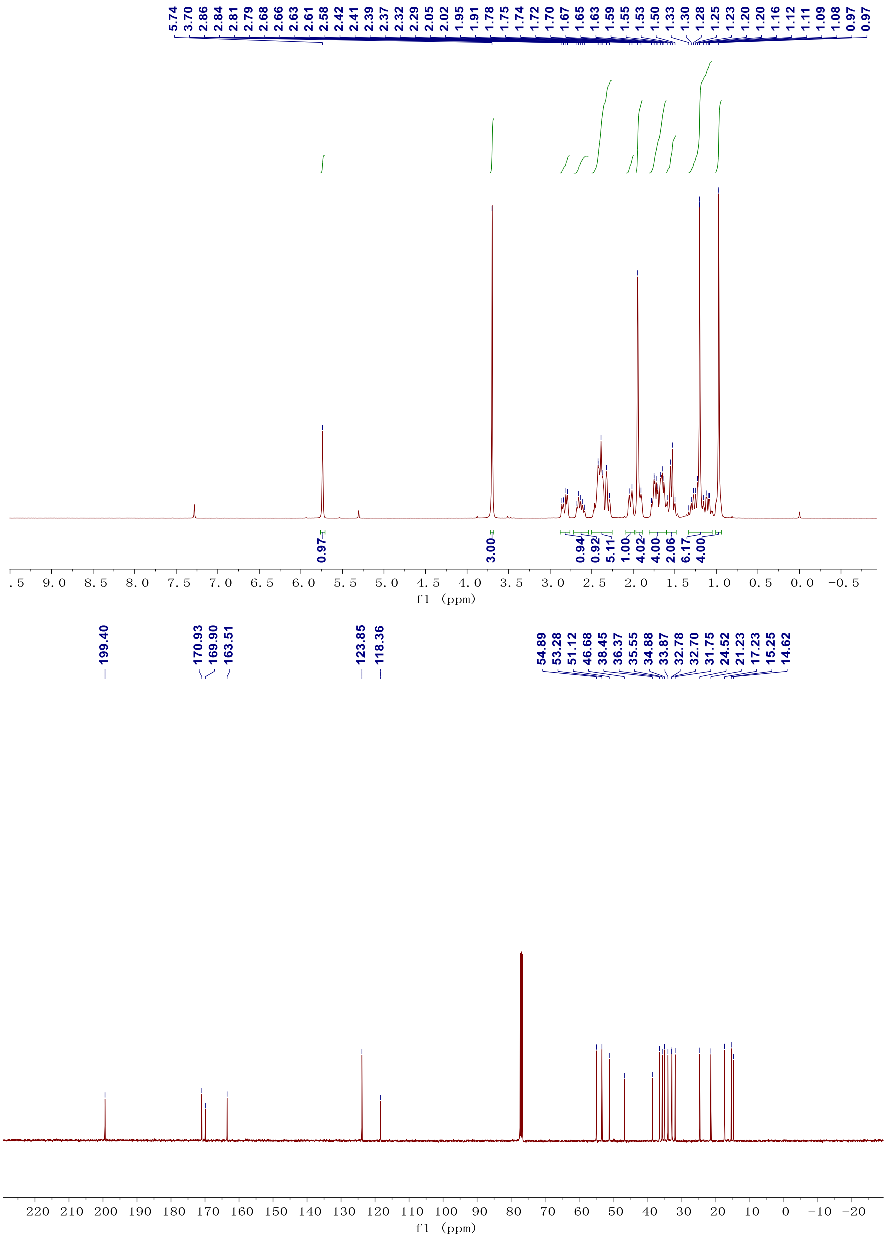

FIGURE S3 NMR spectra of PDCE (**XVI**).

^{1}H NMR (400 MHz, $CDCl_3$) δ 5.74 (s, 1H), 3.70 (s, 3H), 2.83 (dd, J = 19.3, 7.8 Hz, 1H), 2.63 (dt, J = 19.3, 8.9 Hz, 1H), 2.50 – 2.25 (m, 5H), 2.03 (d, J = 13.4 Hz, 1H), 1.95 (s, 4H), 1.78 – 1.63 (m, 4H), 1.60 – 1.48 (m, 2H), 1.33 – 1.05 (m, 6H), 1.01 – 0.94 (m, 4H). ^{13}C NMR (101 MHz, $CDCl_3$) δ 199.4, 170.9, 169.9, 163.5, 123.8, 118.4, 54.9, 53.3, 51.1, 46.7, 38.4, 36.4, 35.6, 34.9, 33.9, 32.8, 32.7, 31.8, 24.5, 21.2, 17.2, 15.2, 14.6.
